# Foreign Correspondence

**Published:** 1870-04

**Authors:** 


### Foreign Correspondence.

K. K. ALLGEMEINEN KRAUKENHAUS,

*Vienna, Jan. 31st, 1870.*

CLINIC OF PROF. HEBRA.

CASE I. Young woman about twenty years of age, has a yellowish incrustation or scab, about as large as a cent, just under the eye, somewhat elevated above the surface of the surrounding parts. The disease is lupus erythematodes. The locality speaks for this disease. She has had it several years. Experiences no pain or itching. We know nothing of the causes of this species of lupus. The microscope in this, as in some other skin diseases, can make no diagnosis for us. It very seldom occurs on children under ten years of age; and ten or more times as often by women as men. It never occurs combined with lupus vulgaris, or other species, but alone.

Has known one patient who had a small patch of lupus e., and while taking a course of arsenic, broke out with lupus e. universalis. The arsenic was abandoned and the lupus vanished. Arsenic was again instituted, and was followed by a new outbreak of the disease. Caustic potash has less influence on this than on the other species of lupus. The application of caustic potash followed immediately by arg. nit., has proven itself to be the speediest method. It sometimes takes months or years to cure this species, hence one must be careful about his prognosis. Treated one young lady six years before effecting a cure. Knew of one case cured simply by washing with soap several times daily. Could destroy this lupus with one application of Arg. Nit. in substance, but it would leave behind a scar not to be wished in the case of a young lady.

Hence, will proceed slower and apply—

℞. Potas. Caustici, Aq. distil., āā partes æquales, with a charpie pencil or brush, and follow it with—

℞. Arg. Nit. Sol., Conc., and cover the part with mercurial plaster. So soon as the parts destroyed fall off, we will repeat the process.

Case II. Lupus Vulgaris.—Patient is a boy five years old. Has a small patch of lupus on each cheek. Will again cauterize with Arg. Nit. in substance, and apply mercurial plaster. Has had three applications and is nearly healed.

Case III. Eczema Capitis. Patient a female of 60 years. The disease covers the head, forehead, ears, and extends to the neck, in the form of yellowish scabs and dry white scales, with a reddish cutis. The exudating stage is nearly passed. Is chlorotic. Did not have her periods until twenty years of age, and they usually continued a week. Is generally a disease of chlorosis. Ordered to smear the parts covered with crusts with oil, and paint the other parts with Ol. Cadini, daily, and wash the parts thoroughly with soap and water before the application.

Case IV. Sycosis.—Male aged 26. Has had the disease eleven years. Is sometimes called mentegra (we often call it Barber's itch). The disease consists in inflammation and suppuration of the hair sacks, and is generally confined to the beard, but sometimes extends to the cilia and hair of the head. The disease occurs under the form of an inflamed hair follicle, which develops into a pustule, which is penetrated by a hair or beard. The skin surrounding is swollen and œdematous in acute cases, and often exudates serum like eczema,* with which it is often confounded and combined. When several of these pustules occur near each other, they often combine and form a yellow scab, and may be mistaken for syphilis. But the removal of the scabs reveals no loss of substance, or but a very slight one, which is not the case with syphilis. The deeper tissues of the skin often become inflamed and give rise to numerous painful abscesses, which leave cicatrices and disfigurements behind, as in this case. The cause of the disease is not settled. Perhaps young hairs may press into the hair sack before the old one is cast off, and thus produce inflammation. The

*I have seen one or two cases of sycosis in Chicago, which had been treated for chronic eczema, syphilis, acne, tetter, and lupus; had taken large quantities of arsenic and other *standard* remedies, with no beneficial results, yet I am convinced they might be easily cured by this simple method, as I have seen so many similar case healed here in six weeks. F.

French school maintain that a parasite is the ætiological moment, but all their attempts in this school to find a parasite have been thus far in vain.

The treatment consists in applying Empl. Diachyl. Comp., q. s. to macerate and remove the crusts and epidermis. The face must be closely shaven every day, and the epulation or extraction of all the hairs perforating pimples or pustules undertaken. After which the ointment may again be applied, spread upon linen cloth. This process must be repeated every day. The treatment in most cases will scarcely exceed from four to six weeks. Persons having a beautiful beard, and not wishing to loose it, may be cured by the above process, omitting the shaving, but the time of treatment will be much increased.

When these diseased hairs are extracted, a new growth will follow them, but when left to die and fall out, they will never be replaced. Patients, when cured, will do well to shave daily for a year, otherwise a return of the disease will be liable to occur. Blepharitis is a disease the same in nature, and may be similarly treated.

Case VI. Syphilis, Scabies, and Eczema.—Female aged 45. House patient. Burrowings of the itch parasite are easily seen on the hands and breasts, and an almost universal eczema produced by scratching and irritation of the skin, while about the shoulders are circular patches of syphilitic ulcers, healed in the centre, leaving white cicatrices. Angina is visible in the pharynx, and the history speaks for a specific disease.

This patient has already had ten sublimate baths, and is improving rapidly. Can cure scabies by sublimate baths alone, but the cure is slower than the one ordinarily instituted for scabies alone. No special treatment is necessary for the eczema, as it will vanish with the cure of the scabies, and the cessation of itching and scratching. The patient remains in the bath two hours daily.

To each bath of simple water is added—

℞. Hydrag. Sublim. Corros.,---------------- ʒij.

Ammon. Hdyro. Chlor.,------------------ ℥ss.

Aqua Fontis,---------------------------- ℥iv.

Case VII. Eczema Capitis.—Female, 22, unmarried. Appearances much the same as in Case III. This is also in this case a disease of chlorosis. Patient is pale, though not badly nourished. Her periods began at eighteen, and now continue from six to eight days; are excessive also in quantity, and irregular. Appetite poor. In such cases there is often a deposit of pigment on the forehead and about the mouth. This disease or form of eczema, frequently occurs in anemic females. Women bearing children often have an outbreak of it after each delivery, which vanishes spontaneously, soon after again becoming pregnant. They have this experience year after year. Earrings are often the cause of eczema, which may spread rapidly over the head and face; hence, should always be removed in cases like this. Some cases will heal with their removal, without other treatment.

Patient ordered to apply oil to the head at night, wash thoroughly with soap and water in the morning, and afterwards apply Ol. Cadini. Also ordered a tonic of iron and quinine, and to visit the clinic again in one week.

Several other cases of scabies followed, of which I may speak in another communication.

Case VIII. Eczema Capitis.—Female aged 30. Head covered with whitish, or whitish-brown scales, with the skin somewhat reddened. Sometimes called pityriasis. She has also had irregular menstruation.

Apply daily—

℞. Tr. Rusci., ---------------------------------- ℥ss.

Alcohol, ---------------------------------- ℥iss.

Ether Sulph., ------------------------------ ℥iss.

Misce.

Case IX. Eczema Manum.—Male, 40. Works in metal foundry, and brings his hands in contact with acidulated waters, etc., which has destroyed the epidermis in many places, while in others they are excoriated and fissured so that he is unable to shut his hands, or to continue his labor.

We might apply the ordinary unguentum diacyli as in cases of eczema on other parts of the body, but we will in this, as in many other similar cases, order him to wear rubber gloves, which will soon render him able to continue his labor, at the same time he continues the treatment. They have the same influence in a great degree as most of the various ointments used in eczema, namely, macerated the epidermis. They should be worn day and night, bound about the wrist to prevent the entrance of air. They should be removed morning and evening, and cleansed with cold water, and the hands thoroughly washed with soap. They are one of the most effectual remedies we possess.

I believe I have mentioned, in one of my previous letters, the great disproportion between the number of students here, and the accommodations for them, in clinics, lectures, etc. The clamor on the part of the students finally became so loud that it resulted in a meeting of the faculty, and the adoption of some new regulations.

It was decided in this meeting that the lecture halls, etc., must be enlarged, or the number of student (about 1500) decreased. The government, however, was severely taxed to build new palatial barracks for soldiers, and to pay the expenses of the King's journey to Egypt, etc., and could not build a new University; therefore the number of students must be diminished.

Professors Hyrtl and Brucke have each from three to five hundred listeners, and only seats for about half that number. So it was decided that each professor should only inscribe as many students as he had seats; moreover, that the price of lectures for foreigners should be doubled, and no more scholarships or free tickets be given. Whereupon the students of Austria proper called a meeting, and petitioned the faculty to consider the Hungarian students, who are in the majority, foreigners, which was impolitic at this time, when so great efforts are being made to affiliate the two countries.

I also hear to-day, that the faculty decided at a late meeting to entirely do away with private courses, given by assistants, as many of them receive a larger salary than the professors. One assistant told me he had made about 5000 guilders, by giving courses in the previous year. In their stead, courses will be given by "private docents," men who have served their time as assistants, and are now practicing in the city.

Much of the above I have simply heard among the students, and may be only rumor; hence will stand subject to correction upon better information.

Opening abscesses and buboes under water, and applying plaster Paris, is being tried here with quite satisfactory results, so far as I can learn. F.

